# An In Situ Oxidative Polymerization Method to Synthesize Mesoporous Polypyrrole/MnO_2_ Composites for Supercapacitors

**DOI:** 10.3390/molecules30010045

**Published:** 2024-12-26

**Authors:** Yan Song, Yangbo Dong, Wei Li, Zhengwen Tan, Pingfei Ma, Guibin Wang, Xuefeng Li

**Affiliations:** 1 Alan G. MacDiarmid Institute, College of Chemistry, Jilin University, 2699 Qianjin Street, Changchun 130012, China; yansong0222@163.com (Y.S.); wgb@jlu.edu.cn (G.W.); 2 State Key Laboratory of Inorganic Synthesis and Preparative Chemistry, Jilin University, 2699 Qianjin Street, Changchun 130012, China; dongyangbo0814@163.com (Y.D.); 15310568206@163.com (Z.T.); cmshmpf@163.com (P.M.); 3 Shanxi Key Laboratory of Coal–Based Value–Added Chemicals Green Catalysis Synthesis, School of Chemistry and Chemical Engineering, Shanxi University, Taiyuan 030006, China; liweichem@sxu.edu.cn

**Keywords:** in situ oxidative polymerization, co–assemble, electrochemical performance

## Abstract

Manganese dioxide (MnO_2_) shows great potential in the field of electrochemical performance. But its poor conductivity, easy dissolution in electrolytes and undesirable ionic accessibility hinder its application. The construction of mesoporous polypyrrole/manganese dioxide (PPy/MnO_2_) composites can effectively alleviate these problems. Herein, an in situ oxidative polymerization method is developed to synthesize mesoporous PPy/MnO_2_ composites. In this method, Pluronic P123 and pyrrole monomers are co-assembled on the surface of MnO_2_. MnO_2_ is used as an oxidation initiator to polymerize pyrrole under acidic conditions and as a substrate for a uniform coating of PPy. The obtained composites, with a large electrochemical effective area, more reaction sites and good structural stability have better capacitor performance (182.8 F g^−1^), higher than MnO_2_ (116.6 F g^−1^) at the same current density. This method provides a meaningful reference for the development of mesoporous PPy/MnO_2_ supercapacitor materials.

## 1. Introduction

Supercapacitors attract significant attention, ascribed to their excellent properties, for example, their capability to deliver high energy, their rapid charge/discharge speed and their long–term cycle stability [1,2]. Manganese dioxide (MnO_2_) is recognized as a potential supercapacitor material because it has a remarkable theoretic specific capacity, a broad voltage window, minimal toxicity and low cost [3,4]. Nevertheless, its poor conductivity and ease of dissolution in neutral electrolytes limit its electrochemical performance [5]. Up to now, many groups have propounded various approaches to tackle these questions, such as combining it with conductive materials [6,7,8], metal cation embedment [9,10] and using the electrodeposition method [11]. In these approaches, the combination of MnO_2_ with conducting materials can effectively improve the electrical conductivity of MnO_2_ and enhance its structural stability, thus increasing the supercapacitor’s capacitance, but the low porosity of the composites inevitably results in lower utilization of the MnO_2_ surface and unsatisfactory ion accessibility [12,13,14].

Mesoporous materials, which have a fairly high specific surface area, can afford more reaction sites, shorten mass transfer distance and facilitate ion transport in active electrode materials, significantly improving electrochemical performance [15,16,17,18,19]. Taking advantage of their high surface area and high porosity, the construction of mesoporous polypyrrole/manganese dioxide (PPy/MnO_2_) composites is an efficient method for improving electrochemical performance by enhancing their electrical conductivity, inhibiting the dissolution of MnO_2_ in neutral electrolytes and increasing the electrochemical effective area and ion accessibility of MnO_2_. However, the relevant literature is poor, and the synthesis of mesoporous PPy/MnO_2_ presents some difficulties. Tong et al. [20] successfully synthesized hollow mesoporous PPy@MnO_2_ composites using mesoporous SiO_2_ as a hard template via a low–temperature redox reaction. But there is the risk of difficult removal of the template with a hard template method. Moreover, Song et al. [21] prepared MnO_2_/PPy composites with typical core–shell structures by combining the redox reactions of KMnO_4_ and MnSO_4_ with the chemical oxidative polymerization of pyrrole in a segmented plug microreactor. This method reduces the size of the material from the micrometer scale to the nanometer scale. But it may be difficult to have control over the reaction kinetics in synthesis systems, which prevents PPy from being efficiently encapsulated on the surface of MnO_2_ [22].

Herein, we demonstrate a facile in situ oxidative polymerization method to synthesize mesoporous PPy/MnO_2_ in an aqueous synthesis system. Pluronic P123 is used as the template to co–assemble with pyrrole monomers to form stable composite micelles in aqueous solutions. The template could be removed through aqueous washing to obtain mesoporous PPy/MnO_2_ without a complicated template removal step. MnO_2_ initiates pyrrole monomer polymerization under acidic conditions and serves as a substrate template to grow pyrrole uniformly on the surface of MnO_2_. This method is an eco–friendly route to synthesize mesoporous PPy/MnO_2_ and provides a new synthetic route for the synthesis of mesoporous composites. The obtained product inhibits the dissolution of MnO_2_ and improves the conductivity and electrochemical effective area of the material. Benefiting from these advantages, mesoporous PPy/MnO_2_ composites show outstanding supercapacitor behavior with a higher specific capacitance of 182.8 F g^−1^ than MnO_2_ (116.6 F g^−1^) at the same current density.

## 2. Results and Discussion

**Scheme 1:**
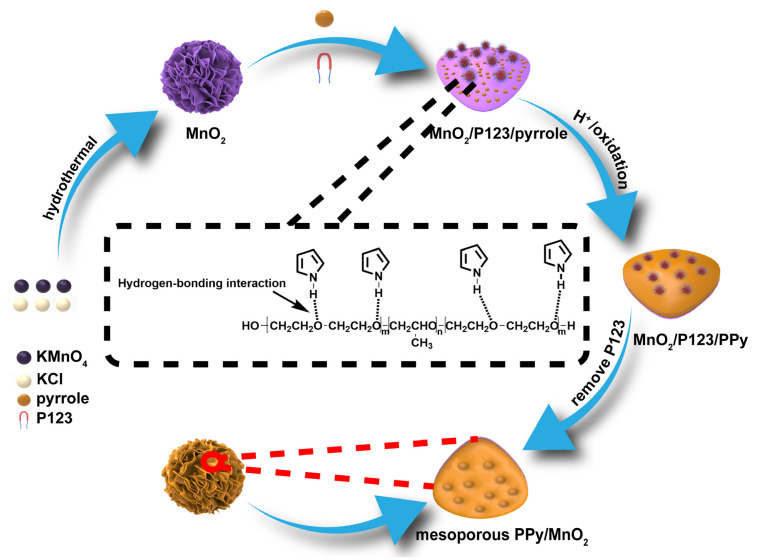
The construction of mesoporous PPy/MnO_2_ composites through a facile in situ oxidative polymerizing process.

Scheme 1 shows the route of formation of the mesoporous polypyrrole/manganese dioxide (PPy/MnO_2_) composites. MnO_2_ was first prepared hydrothermally at 160 °C. KMnO_4_ and KCl were used. The hydrothermal temperature and the concentration of KMnO_4_ have an effect on the morphology of MnO_2_ [23]. Pluronic P123 was assembled into micelles in aqueous solution at 40 °C, followed by the addition of MnO_2_ and pyrrole. Pyrrole monomers were absorbed in the PEO domain of P123 via hydrogen bonding to form stable composite micelles (Figure S1). MnO_2_ works as an oxidation initiator to polymerize pyrrole under acidic conditions (Figure S2) and as a substrate for a uniform coating. Subsequently, mesoporous PPy/MnO_2_ composites with a well-maintained pore structure were obtained by removing P123 through several water and alcohol washes.

**Figure 1:**
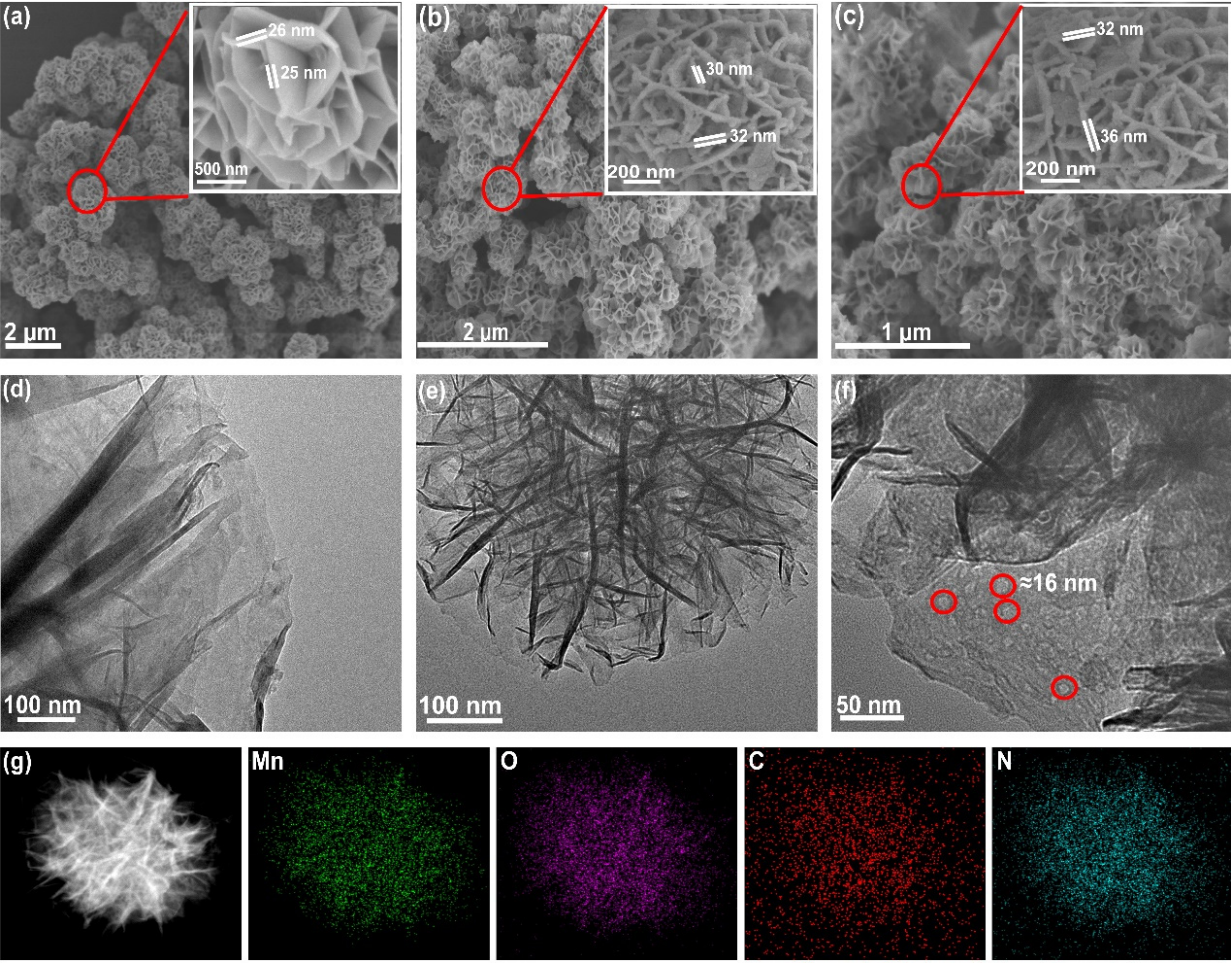
SEM and TEM images: (**a**,**d**) MnO_2_, (**b**,**e**) PPy/MnO_2_ and (**c**,**f**) meso–PPy/MnO_2_–3; (**g**) EDS mapping of meso–PPy/MnO_2_–3. (The red circles in are used to circle the mesopores in the image, it represents where the mesopores are located).

The scanning electron microscope (SEM) and transmission electron microscope (TEM) images (Figure 1a,d) show that the MnO_2_ nanoflowers consist of stacked layers with a width of ≈25 nm. After the introduction of pyrrole, the thickness of the nanosheets increases without any significant change in the structure, and the TEM image shows the nonporous structure (Figure 1b,e). In the presence of P123, the SEM image indicates that the surface of meso–PPy/MnO_2_–3 becomes rough and the TEM image shows the mesoporous structure (Figure 1c,f). Elemental mapping via high–angle annular dark–field scanning transmission electron microscopy (HAADF–STEM) also reveals the homogeneous diffusion of Mn, O, C and N in the meso–PPy/MnO_2_–3 sample (Figure 1g). The impact of P123 concentration (C_P123_) on the porous structure of the samples is further examined with an optimal ratio of MnO_2_ and pyrrole (4:1). As C_P123_ increases from 0 mmol to 0.4 mmol, the porous structure of the sample transforms from a nonporous structure (C_P123_ = 0.07 mmol, labeled as dense–PPy/MnO_2_–1) to a mesoporous structure (C_P123_ = 0.14 mmol, labeled as meso–PPy/MnO_2_–2; C_P123_ = 0.21 mmol, labeled as meso–PPy/MnO_2_–3) and a less porous structure (C_P123_ = 0.28 mmol, labeled as meso–PPy/MnO_2_–4) and a nonporous structure (C_P123_ = 0.4 mmol, labeled as dense–PPy/MnO_2_–5) (Figure S3). Low C_P123_ is unable to reach the crucial micellar concentration, resulting in a nonporous structure [24]. Conversely, high C_P123_ may result in enhanced interactions between micelles, which interferes with their normal function as templates and ultimately affects the structure of the mesoporous material [25].

**Figure 2:**
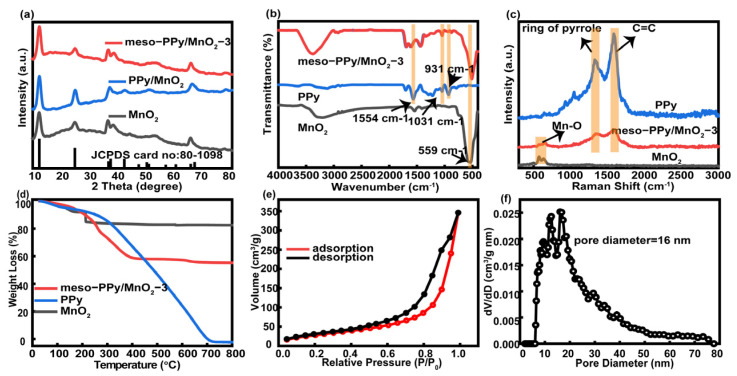
XRD pattern and TG curves of (**a**,**d**) MnO_2_, PPy/MnO_2_ and meso–PPy/MnO_2_–3; FT–IR spectrum and Raman spectrum of (**b**,**c**) MnO_2_, PPy and meso–PPy/MnO_2_–3; and N_2_ adsorption and desorption isotherm and pore size distribution curve of (**e**,**f**) meso–PPy/MnO_2_–3. (The arrows in Figure 2b represents the characteristic peaks of PPy and MnO_2_ in FT–IR spectrum. The arrows in Figure 2c represents the characteristic peaks of PPy and MnO_2_ in Raman spectrum).

The samples were analyzed using various measurement methods. The X–ray diffraction (XRD) pattern of MnO_2_, PPy/MnO_2_ and meso–PPy/MnO_2_–3 is shown in Figure 2a. Four typical peaks at 2θ angles of 12.52°, 25.14°, 37.28° and 67.9° correspond to the (001), (002), (–111) and (114) crystalline surfaces of MnO_2_ (JCPDS card no: 80–1098, a = 5.149 Å, b = 2.843 Å and c = 7.716 Å). The Fourier–Transform Infrared (FT–IR) spectrum of the fabricated PPy, MnO_2_ and meso–PPy/MnO_2_–3 composite is shown in Figure 2b. For MnO_2_, the peak between 400–700 cm^−1^ is attributed to the Mn–O bond [26]. The symmetrical stretching vibration of the C=C bond in PPy rings at 1554 cm^−1^, N–H in–plane deformation vibration of the PPy at 1031 cm^−1^ and C–H out–of–plane vibrations at 931 cm^−1^ are the characteristic peaks of PPy [27,28]. The spectrum of meso–PPy/MnO_2_–3 shows the typical peaks of PPy and MnO_2_, but the intensity of the peaks is significantly weakened and the position of the peaks is shifted. For example, the characteristic peak of MnO_2_ is red–shifted, which may be due to the hydrogen bonding between the oxygen atom of Mn–O in MnO_2_ and the hydrogen atom of N–H in PPy. The blue shift of the characteristic peak of PPy may be due to the effect of electron transfer between PPy and MnO_2_ [29].

The Raman spectra of the samples were obtained. Two distinct spectral bands near 1355–1569 cm^−1^ and the spectral bands near 505–575 cm^−1^ are attributed to PPy and MnO_2_, separately (Figure 2c). In addition, the distinctive peaks of MnO_2_ and PPy were found in meso–PPy/MnO_2_–3, indicating the presence of coexisting MnO_2_ and PPy. In order to confirm the content of meso–PPy/MnO_2_–3, thermogravimetric (TG) tests were also carried out (Figure 2d). The weight loss at around 200 °C of meso–PPy/MnO_2_–3 was about 15 wt.%, attributed to the elimination of adsorbed water on the surface [30,31,32]. Further weight loss from 200 °C to 500 °C correlates with the decomposition of the PPy chains [30], suggesting that the weight loss of PPy is about 25 wt.%, which is in agreement with the CHN data (Table S1). The N_2_ adsorption and desorption curves of mesoporous PPy/MnO_2_–3, which has numerous mesopores (Figure 2e), show that it has a typical type IV curve and a very obvious H3–type hysteresis loop. The Brunauer–Emmett–Teller surface area of meso–PPy/MnO_2_–3 was 116 m^2^ g^−1^ and the average pore size was assessed as 16 nm (Figure 2f), which agrees well with the TEM result (Figure 1f).

**Figure 3:**
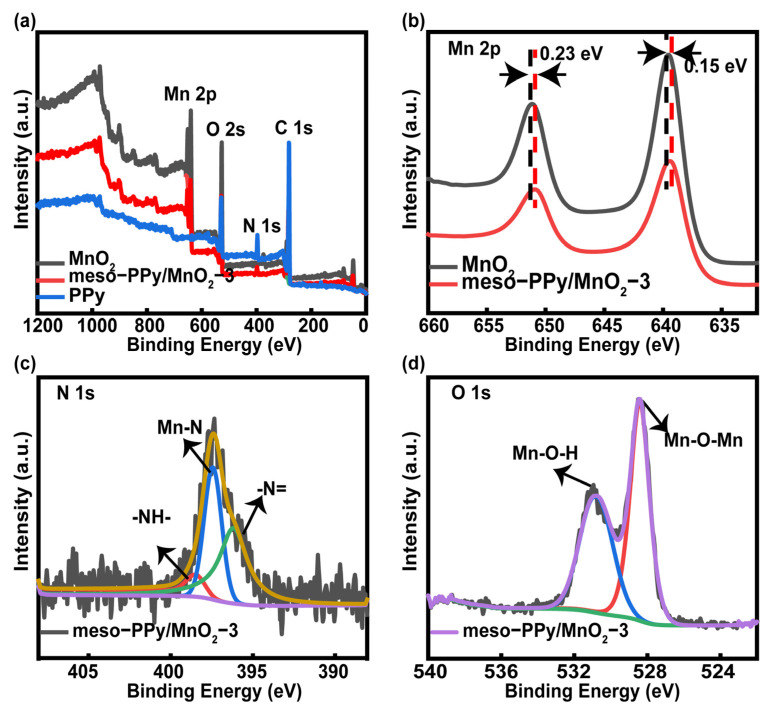
XPS spectrum of (**a**) MnO_2_, PPy and meso–-PPy/MnO_2_-3 and high-resolution spectrum of (**b**) Mn in MnO_2_ and meso-PPy/MnO_2_-3, (**c**) N in meso-PPy/MnO_2_-3 and (**d**) O in meso-PPy/MnO_2_-3.

We used X–ray photoelectron spectroscopy (XPS) to probe the chemical properties of MnO_2_, PPy and mesoporous PPy/MnO_2_–3. MnO_2_ has three distinct peaks at 285, 529 and 640 eV corresponding to C 1s, O 1s and Mn 2p. PPy has three characteristic peaks at 285, 400 and 529 eV corresponding to C 1s, O 1s and N 1s. There are four distinct peaks at 285, 400, 529 and 640 eV in mesoporous PPy/MnO_2_–3, corresponding to C 1s, N 1s, O 1s and Mn 2p, which indicates the successful complexation of MnO_2_ with PPy (Figure 3a). For the high–resolution Mn 2p spectrum of mesoporous PPy/MnO_2_–3 (Figure 3b), the peak centered at 639.2 eV is assigned to Mn 2p_3/2_ and 650.9 eV is assigned to Mn 2p_1/2_. The spin energy separation between the Mn 2p_3/2_ and Mn 2p_1/2_ peaks is 11.7 eV, which confirms the existence of Mn^4+^ in the sample, nearly the same as the reported values [33]. Figure 3b also shows that the Mn 2p_1/2_ and Mn 2p_3/2_ peaks of meso–PPy/MnO_2_–3 (ΔE = 0.23 eV and 0.15 eV) show negative shifts with respect to pure MnO_2_, indicating the increase in electronic density of the Mn center offered by PPy [34]. The XPS peak of N 1s was decomposed to three Gaussian peaks. The binding energy at 398.2 eV (–N=) corresponds to an imine–like structure and that at 399.3 eV (–NH–) corresponds to a neutral-like structure, whereas the peak at 398.8 eV is the Mn–N bond because there is a strong interaction force between the MnO_2_ and the PPy. The Mn–N bond can efficiently solve the problem of the dissolution of manganese in electrolytes [35]. Figure 3d shows the high–resolution O 1s spectrum with two characteristic peaks at 528.5 eV and 530.8 eV, corresponding to Mn–O–Mn and Mn–O–H of the mesoporous PPy/MnO_2_–3 composite.

**Figure 4:**
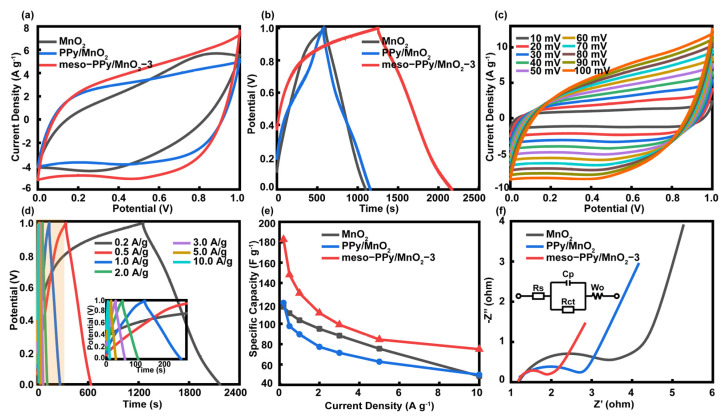
CV and charge–discharge curves of (**a**,**b**) MnO_2_, PPy/MnO_2_ and meso–PPy/MnO_2_–3 and (**c**,**d**) meso–PPy/MnO_2_–3; (**e**,**f**) rate capability and Nyquist plots of MnO_2_, PPy/MnO_2_ and meso–PPy/MnO_2_–3.

Meso–PPy/MnO_2_, which has a high specific surface area and mesoporous channels will become a promising electrode material in the future. The tri–electrode system was assembled to perform electrochemical tests to assess the electrochemical properties. The system employs meso–PPy/MnO_2_ as the operating electrode, Ag/AgCl as the standard electrode and Pt as the counter electrode. At 50 mV s^−1^, the cyclic voltammetry (CV) curves in Figure 4a of PPy/MnO_2_ and meso–PPy/MnO_2_–3 are regular rectangles, suggesting that they have better conductivity. The CV curve of MnO_2_ has a prominent spindle shape at both ends due to its deficient electrical properties, limited ionic transport capacity and the ease of dissolution of MnO_2_ in neutral aqueous solution (pH = 7). The integral area of the cyclic voltammetry curve of meso–PPy/MnO_2_–3 is larger than that of PPy/MnO_2_ and MnO_2_, which can be ascribed to the existence of mesopores in meso–PPy/MnO_2_–3 [36]. The mesoporous structure permits rapid ion transport, improves the utilization of MnO_2_ and enhances the electrochemical properties [37,38]. At 100 mV s^−1^, the well-maintained shape of the CV curve indicates the stable electrochemical properties of meso–PPy/MnO_2_–3 (Figure 4c).

The galvanostatic charge/discharge (GCD) measurement of meso–PPy/MnO_2_–3 was performed at different current densities with potentials ranging from 0 to 1.0 V. At 0.2 A g^−1^, the GCD curve of meso–PPy/MnO_2_–3 was seen to have a triangular shape, which indicates that adsorption–desorption is highly reversible during charging and discharging (Figure 4b). The CV and GCD curves of the other composites are exhibited in Figure S5. Among them, the optimum capacitance of the meso–PPy/MnO_2_–3 electrode is 182.8 F g^−1^, which is better than PPy/MnO_2_ (120.52 F g^−1^) and MnO_2_ (116.6 F g^−1^) at 0.2 A g^−1^ current density. The specific capacitances of meso–PPy/MnO_2_–3 are 148.1, 130 and 110.64 F g^−1^ when the current density is changed in the range from 0.5 to 2 A g^−1^ (Figure 4d). At 10 A g^−1^, the capacitance of meso–PPy/MnO_2_–3 (79.8 F g^−1^) exceeded that of PPy/MnO_2_ and MnO_2_, indicating the improved capacity and rate performance of meso–PPy/MnO_2_–3 (Figure 4e).

We performed electrochemical impedance tests to study the charge transfer resistance (Rct) and ion transport ability of the meso–PPy/MnO_2_ composites (Figure 4f). Due to the nature of the EIS equivalent circuit, the equivalent circuit we used is a commonly employed equivalent circuit model. This model consists of a resistor, a constant phase element (CPE) and a Warburg element (W) [39]. At high frequencies, the Nyquist curve manifests an arc, and the Nyquist curve manifests a spike at low frequencies. The arc represents the charge transfer resistance (Rct), and the spike represents the diffusion process. The Nyquist curve of meso–PPy/MnO_2_–3 is approximately linear with the y–axis, which confirms that meso–PPy/MnO_2_–3 has good diffusive properties and ideal capacitance properties. It also explains the enhanced performance of meso–PPy/MnO_2_–3. The electrochemical impedance spectra are also used to explore the cause of the increase in the specific capacitance of meso–PPy/MnO_2_–3. Based on the radii of the arcs in Figure 4f and the auxiliary infographic Figure S7, the charge transfer resistance magnitudes can be obtained as follows: meso–PPy/MnO_2_–3 (0.53 Ω) < meso–PPy/MnO_2_–4 (0.81 Ω) ≈ dense–PPy/MnO_2_–5 (0.82 Ω) < meso–PPy/MnO_2_–2 (1.15 Ω) < dense–PPy/MnO_2_–1 (1.16 Ω) < PPy/MnO_2_ (1.71 Ω) < MnO_2_ (1.98 Ω). The Rct of meso–PPy/MnO_2_–3 is the lowest among the other samples. Furthermore, the specific capacitance of the meso–PPy/MnO_2_–3 can be maintained at 77.6% after 1000 cycles at 0.5 A g^−1^, higher than MnO_2_ (67.8%) and PPy (59%) (Figure S6a–d). Meso–PPy/MnO_2_–3 exhibits the best capacitance, which may be assigned to the enhanced properties of conductivity and the mesoporous structure of the composite with polypyrrole. These results indicate that the meso–PPy/MnO_2_–3 holds particular promise as a supercapacitor material.

As can be seen in Table 1, the specific capacitance of meso–PPy/MnO_2_–3 in this work is comparable or higher to previous reports, indicating a further improvement in its electrochemical properties. There is still room for optimization in terms of pore size adjustment and pore-directing agent selection. This study provides a new method for the facile synthesis of meso–PPy/MnO_2_ composites, which can also be used for the synthesis of other mesoporous composites.

## 3. Materials and Methods

### 3.1. Preparation of MnO_2_

Briefly, 3 mmol of KMnO_4_ and 1 mmol of KCl were added to 40 mL of deionized water under magnetic stirring to form a homogeneous solution. Then, the solution was transferred to a Teflon–lined stainless–steel autoclave and kept at 160 °C for 12 h. A brown color precipitate was obtained and centrifuged, and the precipitate was washed with deionized water several times followed by ethanol and finally dried at 60 °C overnight.

### 3.2. Preparation of Mesoporous PPy/MnO_2_

In a typical synthesis procedure of mesoporous PPy/MnO_2_, a certain amount of Pluronic P123 was added to 100 mL of deionized water under magnetic stirring to form a stable solution under the condition of a 40 °C water bath. Then, 1.2 mmol of the as-prepared MnO_2_ powder and 0.3 mmol of pyrrole were dispersed in the mentioned solution. After 1 h, a certain volume of HCl (0.06 M) was added under magnetic stirring. As pyrrole polymerized around MnO_2_, the brownish suspension changed its color into a dark suspension. The in situ polymerization reaction proceeded for 4 h. The samples were labeled as PPy/MnO_2_ without P123, dense–PPy/MnO_2_–1 with the amount of 0.07 mmol P123, meso–PPy/MnO_2_–2 with the amount of 0.14 mmol P123, meso–PPy/MnO_2_–3 with the amount of 0.21 mmol P123, meso–PPy/MnO_2_–4 with the amount of 0.28 mmol P123 and dense–PPy/MnO_2_–5 with the amount of 0.4 mmol P123.

### 3.3. Material Characterization

X–ray diffraction (XRD) patterns were collected on a Bruker D8 Advance X–ray diffractometer with monochromatic Cu Kα irradiation. Scanning electron microscopy (SEM) images were taken on a HITACHI SU8020 scanning electron microscope with an accelerating voltage of 3 kV. Transmission electron microscopy (TEM) images were obtained on an FEI Tecnai G2 F20S-Twin D573 field emission microscope at 200 kV. The surface area and pore size of the samples were measured via N_2_ physisorption at 77 K on a NOVA 4200e. All samples were measured after degassing at 100 °C for 6 h. X–ray photoelectron spectroscopy (XPS) measurements were taken on an ESCALAB250 system with a monochromatic X–ray source (Al Kα hv = 1486.6 eV). FT–IR spectra were performed within the 400–4000 cm^−1^ region using a Bruker IFS 66 V/S FT–IR spectrometer with KBr pellets. The weight loss of the samples was measured using a thermogravimetric analyzer (TGA Q50) in N_2_ with a heating rate of 10 °C min^−1^. The Raman spectra of the samples were analyzed on a Renishaw inVia Raman microscope. The optical photographs in this paper were taken with a smartphone (12, Xiaomi, Beijing, China).

### 3.4. Electrochemical Measurements

Electrodes for the supercapacitors were fabricated by mixing 70 wt.% active materials with 20 wt.% carbon black and 10 wt.% polytetrafluoroethylene (PTFE) binder in NMP to make a homogeneous slurry. The slurry was then brush–coated onto Ni foams (1 cm^2^) and pre–dried at 60 °C overnight. Finally, the electrodes were uniaxially pressed under 2 MPa. The mass loading of the active materials was about 3.0 mg cm^−2^. Electrochemical measurements were carried out on a CHI 660B electrochemical station in a three–electrode configuration. The active materials on Ni foam, a platinum chip and an Ag/AgCl electrode were used as the working electrode, counter electrode and reference electrode, respectively. Cyclic voltammetry (CV) and galvanostatic charging/discharging techniques were employed to study the electrochemical properties in a 1 M Na_2_SO_4_ solution of the electrolyte at room temperature. Electrochemical impedance spectroscopy (EIS) was conducted in the frequency range between 100 K Hz and 10 m Hz with the perturbation amplitude of 5 mV versus the open-circuit potential.

## 4. Conclusions

In this work, meso–PPy/MnO_2_ composites were successfully fabricated via a facile in situ oxidative polymerization method. This method can effectively control the reaction kinetics of pyrrole polymerization and solve the problem of the inhomogeneous coating of PPy. This synthesis method also provides a new route for the synthesis of mesoporous composites. The introduction of a mesoporous structure in PPy/MnO_2_ materials increases the number of reaction sites and improves the surface utilization of MnO_2_, which confirms the feasibility of the rational design of electrode materials for electrochemical capacitors.

## Figures and Tables

**Table 1:** A summary of the specific capacitance of the MnO_2_ and PPy/MnO_2_ composite electrode materials for supercapacitors in the literature.

Electrode Materials	Electrolyte	Specific Capacitance (F/g)	Reference
PPy/MnO_2_	1M Na_2_SO_4_	109	[21]
PPy/MnO_2_	1M Na_2_SO_4_	125	[40]
PPy/MnO_2_	1M Na_2_SO_4_	141.6	[41]
PPy/NwMnO_2_	0.1M H_2_SO_4_	203	[42]
MnO_2_/CNT/G	1M Na_2_SO_4_	120	[43]
MnO_2_	1M Na_2_SO_4_	170	[44]
Meso–PPy/MnO_2_–3	1M Na_2_SO_4_	182.8	This work

## Data Availability

The original contributions presented in this study are included in the article; further inquiries can be directed to the corresponding author.

## Supplementary Materials

The following supporting information can be downloaded at: www.mdpi.com/article/10.3390/molecules29010045/s1, Figure S1 FT–IR spectrum of Pluronic P123, pyrrole and P123–pyrrole. Figure S2 (a–d) Images of MnO_2_ supernatant with alkali after 10 min, 20 min, 30 min, and 60 min, (e–h) images of meso–PPy/MnO_2_ centrifuged supernatant with alkali after 10 min, 20 min, 30 min, and 60 min. Figure S3 (a,b) TEM images of dense–PPy/MnO_2_–1, (c,d) TEM images of meso–PPy/MnO_2_–2, (e,f) TEM images of meso–PPy/MnO_2_–4, (g,h) TEM images of dense–PPy/MnO_2_–5. Figure S4 XRD pattern of dense–PPy/MnO_2_–1, meso–PPy/MnO_2_–2, meso–PPy/MnO_2_–4, dense–PPy/MnO_2_–5 and PPy. Figure S5 CV curves and charge–discharge curves of (a,b) dense–PPy/MnO_2_–1, (c,d) meso–PPy/MnO_2_–2, (e,f) meso–PPy/MnO_2_–4, (g,h) dense–PPy/MnO_2_–5. Figure S6 Cyclic stability of (a) meso–PPy/MnO_2_–3, (b) MnO_2_ and (c) PPy, and the capacitance retention % vs. Cycle number of (d) meso–PPy/MnO_2_–3, MnO_2_ and PPy. Figure S7 Nyquist plots of dense–PPy/MnO_2_–1, meso–PPy/MnO_2_–2, meso–PPy/MnO_2_–4 and dense–PPy/MnO_2_–5. Table S1. CHN data.
