# Supplementary material for: An In Situ Oxidative Polymerization Method to Synthesize Mesoporous Polypyrrole/MnO2 Composites for Supercapacitors
Source: Molecules. 2024 Dec 26;30(1):45. doi: 10.3390/molecules30010045 (PMC11720845; doi:10.3390/molecules30010045)
Supplement: Supplementary file 1 [file molecules-30-00045-s001.zip › molecules-3380888-supplementary.pdf]

An In Situ Oxidative Polymerization Method to Synthesize Mesoporous Polypyrrole/MnO_2 Composites for Supercapacitors

Yan Song [1,2,†], Yangbo Dong [2,†], Wei Li [3], Zhengwen Tan [2], Pingfei Ma [2], Guibin Wang [1] and Xuefeng Li [1,*]

[1] Alan G. MacDiarmid Institute, College of Chemistry, Jilin University, 2699 Qianjin Street, Changchun 130012, China
[2] State Key Laboratory of Inorganic Synthesis and Preparative Chemistry, Jilin University, 2699 Qianjin Street, Changchun 130012, China
[3] Shanxi Key Laboratory of Coal−Based Value−Added Chemicals Green Catalysis Synthesis, School of Chemistry and Chemical Engineering, Shanxi University, Taiyuan 030006, China
* Correspondence: xuefengli@jlu.edu.cn; Tel.: +86−13578926668
† These authors contributed equally to this work.

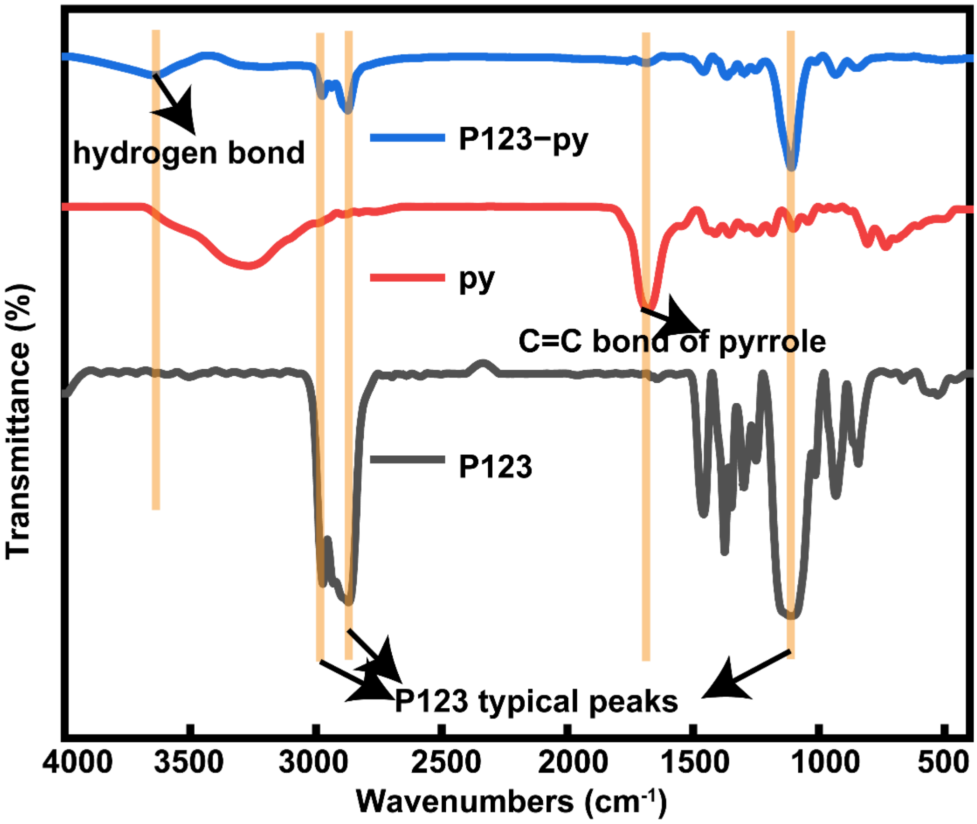

Figure S1. FT−IR spectrum of Pluronic P123, pyrrole and P123−pyrrole.

Note: FT−IR spectrum explains the formation of the hydrogen bond with characteristic peak 3624 cm^{-1}.

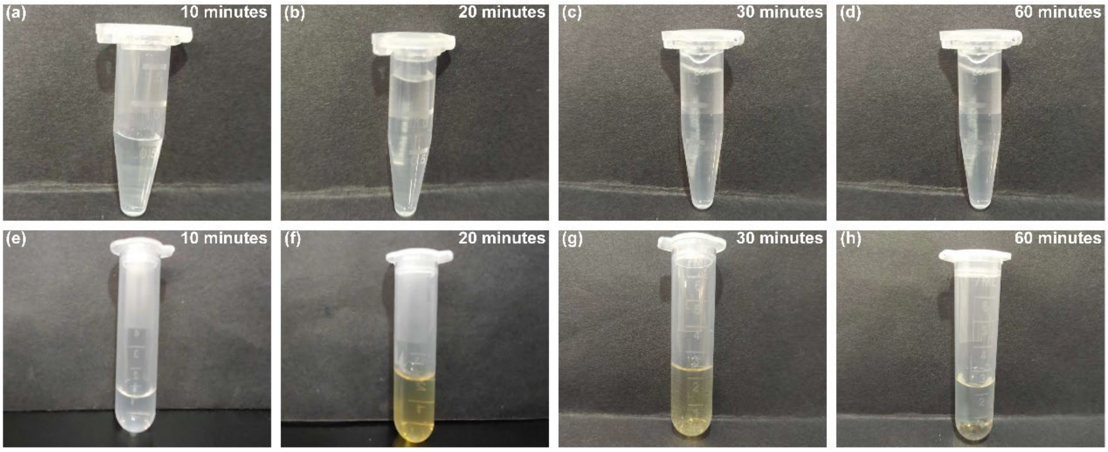

Figure S2. (a−d) Images of MnO_2 supernatant with alkali after 10 minutes, 20 minutes, 30 minutes, and 60 minutes, (e−h) images of meso−PPy/MnO_2 centrifuged supernatant with alkali after 10 minutes, 20 minutes, 30 minutes, and 60 minutes.

Note: Images of Figure S2 confirm the mechanism of synthesizing meso−PPy/MnO_2. MnO_2 is soaked, stirred and centrifuged in acid solution. An excess of alkaline solution is added to the centrifuged supernatant and there is no significant change in the supernatant after 60 minutes, which proves that MnO_2 is not soluble in acid conditions. The same operation is true for the centrifuged supernatant of meso−PPy/MnO_2. It can be found that the supernatant turns yellow and then produces a brown precipitate, which proves the presence of Mn^{2+} ions in the solution. The mechanism by which MnO_2 can polymerize the pyrrole in acidic solutions is verified.

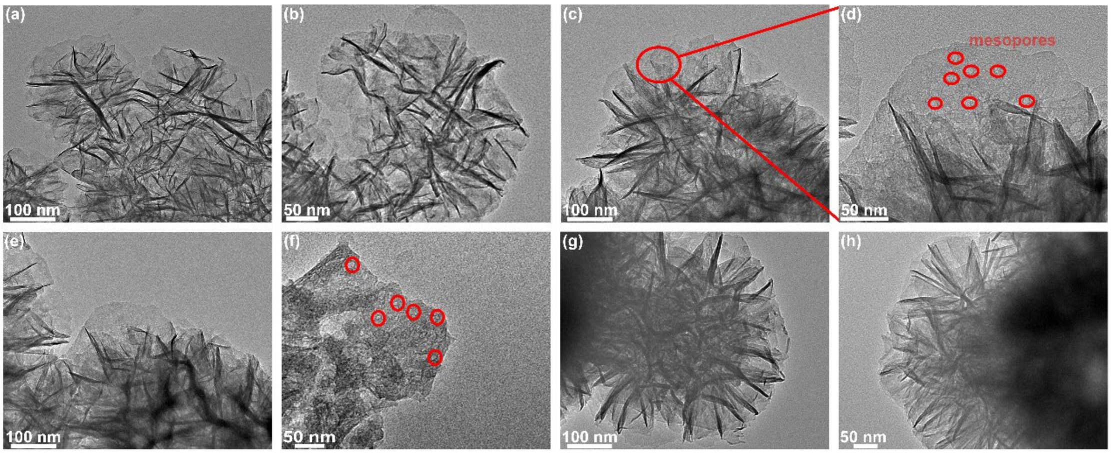

Figure S3. (a−b) TEM images of dense−PPy/MnO_2−1, (c−d) TEM images of meso−PPy/MnO_2−2, (e−f) TEM images of meso−PPy/MnO_2−4, (g−h) TEM images of dense−PPy/MnO_2−5.

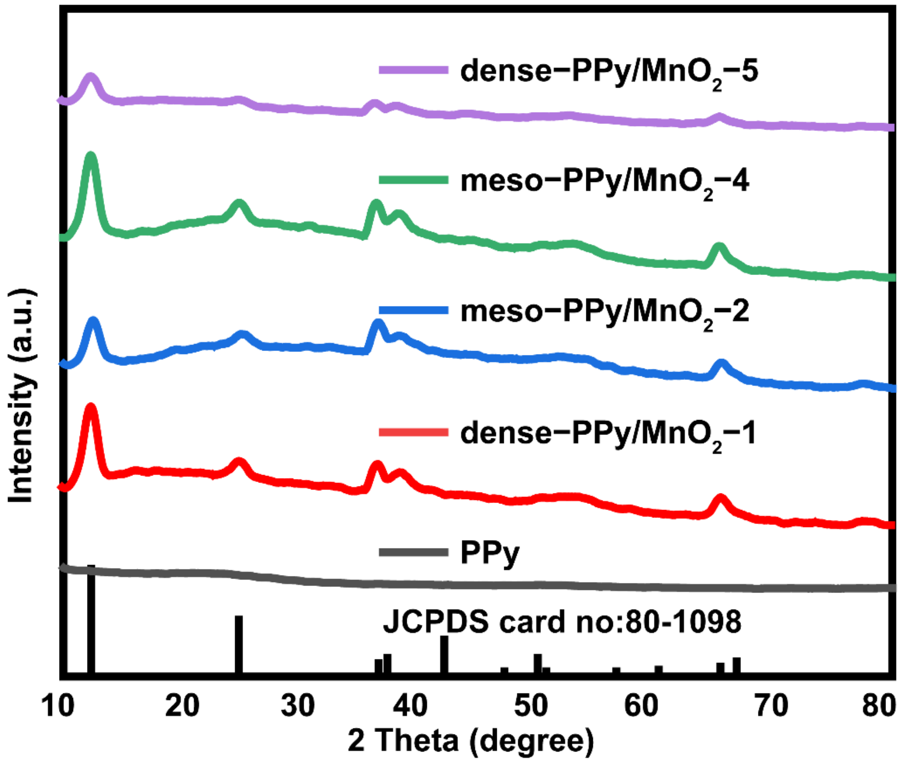

Figure S4. XRD pattern of dense−PPy/MnO_2−1, meso−PPy/MnO_2−2, meso−PPy/MnO_2−4, dense−PPy/MnO_2−5 and PPy.

Note: The XRD pattern suggests that other composites also belong to the structure of MnO_2 (JCPDS card no: 80-1098, a = 5.149 Å, b = 2.843 Å and c = 7.716 Å). The broad peak of PPy indicates its recognized amorphous crystalline phase after polymerization. The disappearance of its broad peak in composites might be attributed to the low content of PPy.

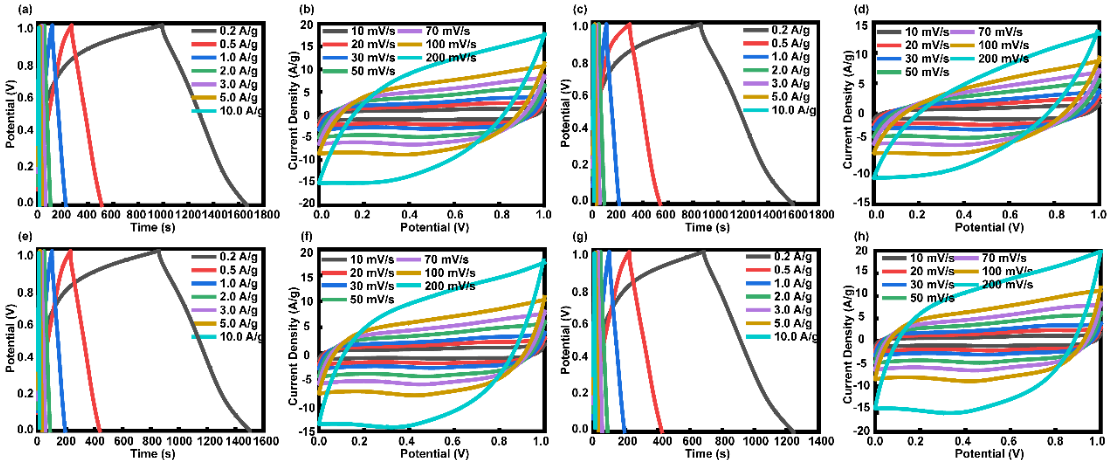

Figure S5. CV curves and charge−discharge curves of (a, b) dense−PPy/MnO_2−1, (c, d) meso−PPy/MnO_2−2, (e, f) meso−PPy/MnO_2−4, (g, h) dense−PPy/MnO_2−5.

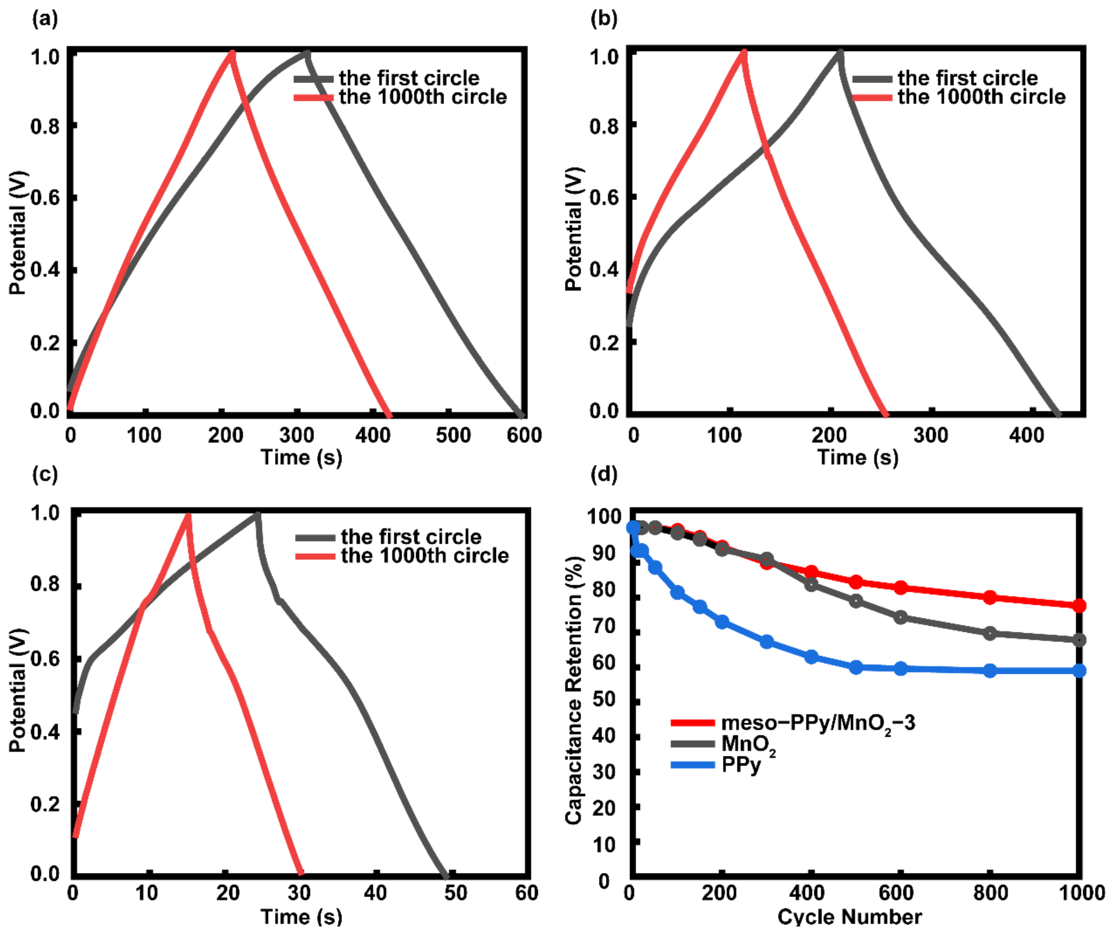

Figure S6. Cyclic stability of (a) meso−PPy/MnO_2−3, (b) MnO_2 and (c) PPy, and the capacitance retention % vs. Cycle number of (d) meso−PPy/MnO_2−3, MnO_2 and PPy.

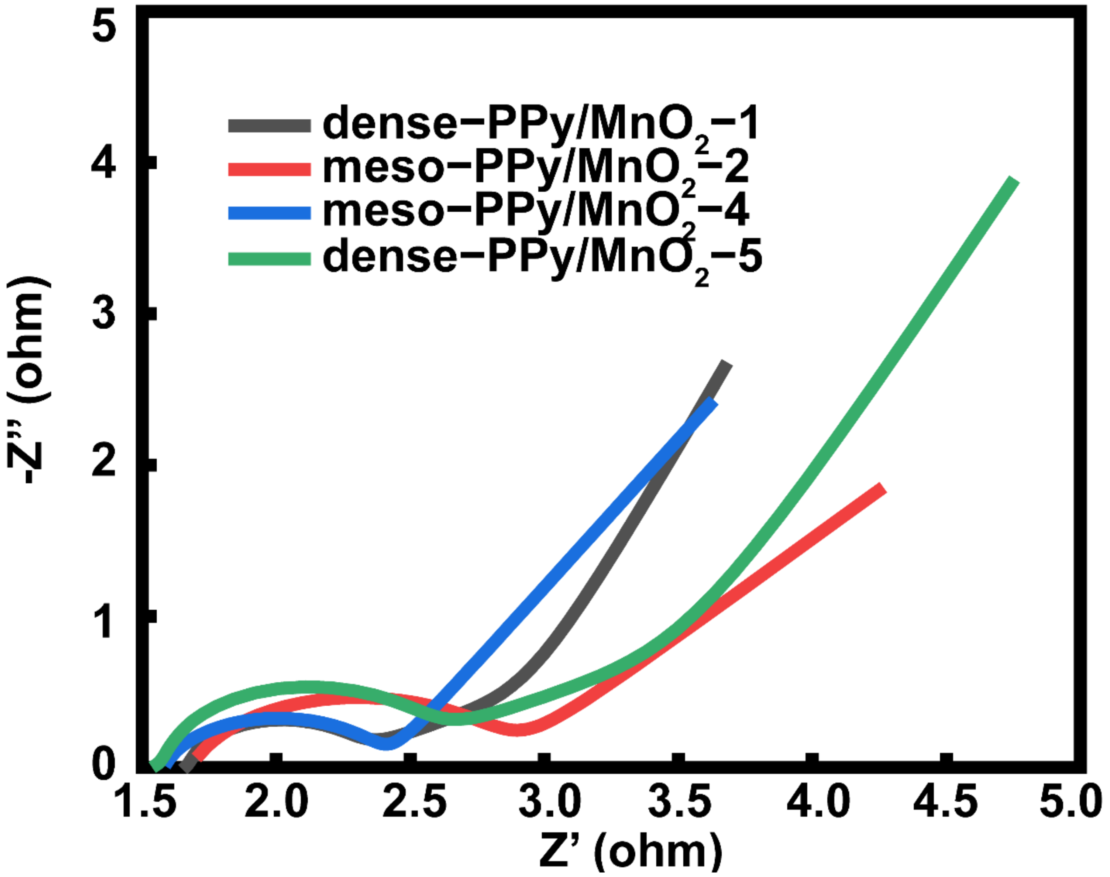

Figure S7. Nyquist plots of dense−PPy/MnO_2−1, meso−PPy/MnO_2−2, meso−PPy/MnO_2−4 and dense−PPy/MnO_2−5.

Table S1. CHN data.

Sample	C (%)	H (%)	N (%)
MnO_2	0.59	0.58	0
PPy/MnO_2	21.20	1.20	5.08
meso−PPy/MnO_2−3	22.06	2.47	3.00
PPy	57.86	3.41	16.42
